# Electronic Science Games Used to Enhance Cognitive Ability: Opinion of Design From Personalization and Adaptation

**DOI:** 10.3389/fnagi.2021.789547

**Published:** 2021-11-12

**Authors:** Dong Wen, Wang Yao, Jian Xu, Shaochang Wang, Yingzhu Zhong, Hongqian Chen, Xianling Dong, M. Iqbal Saripan, Yanhong Zhou

**Affiliations:** ^1^Institute of Artificial Intelligence, University of Science and Technology Beijing, Beijing, China; ^2^School of Information Science and Engineering, Yanshan University, Qinhuangdao, China; ^3^Department of Biomedical Engineering, Chengde Medical University, Chengde, China; ^4^Faculty of Engineering, Universiti Putra Malaysia, Serdang, Malaysia; ^5^School of Mathematics and Information Science and Technology, Hebei Normal University of Science and Technology, Qinhuangdao, China

**Keywords:** electronic science game, cognitive ability, personalization, adaptation, enhance

## Introduction

In recent years, more and more cognitive scientists have incorporated personalized and adaptive elements into the design of cognitive training games. From the perspective of personalization, some researchers allow game players to choose roles, scenes or personalized cognitive training tasks according to their own preferences to achieve personalized game design (Reategui et al., 2006; Wei, 2009; Hollingdale and Greitemeyer, 2013; Lin et al., 2013; Li et al., 2016; Nagle et al., 2016; Orji et al., 2017; Orji and Moffatt, 2018; Soares et al., 2018; Waltemate et al., 2018; Zibrek et al., 2018; González et al., 2019; Knutas et al., 2019; Troussas et al., 2019). There are also some researchers from the adaptive point of view, by dynamically changing the parameters in the game, automatically adapting to the player's game difficulty, dynamically generating new content and other methods to achieve the adaptive design of the game (Carro et al., 2002; Hunicke, 2005; Togelius et al., 2010; Johnson et al., 2014; Li et al., 2014; Yannakakis and Togelius, 2015; Shaker et al., 2016; Schadenberg et al., 2017; Soler-Dominguez et al., 2017; Ashish et al., 2018; Lopes et al., 2018; Shi and Chen, 2018; Souza et al., 2018; Denisova and Cairns, 2019; Dey et al., 2019; Hendrix et al., 2019; Liang et al., 2019; Pan et al., 2019; Papadimitriou et al., 2019; Peng et al., 2019; Plass et al., 2019; Sepulveda et al., 2019). Relevant research showed that adding personalized design to electronic science games for improving cognitive abilities could enhance the cognitive training experience of gamers, stimulate their interest in cognitive training, and better enhance the training experience and cognition ability of gamers (Reategui et al., 2006; Wei, 2009; Hollingdale and Greitemeyer, 2013; Lin et al., 2013; Li et al., 2016; Nagle et al., 2016; Orji et al., 2017; Orji and Moffatt, 2018; Soares et al., 2018; Waltemate et al., 2018; Zibrek et al., 2018; González et al., 2019; Knutas et al., 2019; Troussas et al., 2019); adding adaptive design to electronic science games used to improve cognitive ability, which can match the player's level with the difficulty of the game, so that gamers can obtain the best training effect (Carro et al., 2002; Hunicke, 2005; Togelius et al., 2010; Johnson et al., 2014; Li et al., 2014; Yannakakis and Togelius, 2015; Shaker et al., 2016; Schadenberg et al., 2017; Soler-Dominguez et al., 2017; Ashish et al., 2018; Lopes et al., 2018; Shi and Chen, 2018; Souza et al., 2018; Denisova and Cairns, 2019; Dey et al., 2019; Hendrix et al., 2019; Liang et al., 2019; Pan et al., 2019; Papadimitriou et al., 2019; Peng et al., 2019; Plass et al., 2019; Sepulveda et al., 2019). From the perspective of personalization and adaptability, this article systematically discusses the research status and design methods of electronic science games to enhance cognitive ability, as well as the advantages and challenges of personalized and adaptive design in electronic science games.

## The Study Value of Electronic Science Games with Personalization and Adaptation

In recent years, a series of studies based on brain science methods have shown that electronic science games have the effect of promoting cognitive enhancement and that individual differences in game players will make them obtain different effects in cognitive training (Shang and Zhang, 2017). Therefore, in order to allow gamers to obtain the best training results, more and more researchers are beginning to design games from the perspective of personalization and adaptation, and prove the advantages of personalized and adaptive games through experiments.

In 2019, Ahmed Tlili et al. divided 51 learners into a control group and an experimental group, and they learned through the non-personalized version and the personalized version of the game, respectively (Tlili et al., 2019). The experimental results showed that personalized educational games not only reduced the cognitive burden of learners, but also allowed learners to show higher perceived usefulness and willingness to use. In the same year, Troussas et al. conducted research on personalized games and showed that integrating personalization and collaboration into mobile game learning could further help higher education students to improve their knowledge and cognition level (Troussas et al., 2019).

In 2013, Sampayo-Vargas et al. began to explore adaptive design. They compared adaptive difficulty adjustment games and non-adaptive difficulty adjustment games. The results showed that players who played adaptive games had achieved better results (Sampayo-Vargas et al., 2013).

In 2014, Montani developed a new adaptive game, and the results showed that adaptive games can improve the cognitive ability of game players (Montani et al., 2014). Recently, Daghestani et al. developed a gamification learning system that combines gamification, classification and adaptive technologies. The results showed that the adaptive gamification learning system had a positive impact on students' learning participation and academic performance (Daghestani et al., 2020).

In summary, electronic science games incorporating personalized and adaptive elements have a significant positive effect on increasing player participation, reducing the burden on players in the game, and achieving game training goals.

## Personalization and Adaptation Design of Electronic Science Games

With the continuous development of cognitive science and the video game industry, electronic science games for cognitive enhancement have been sought after. However, how to design such games is the core issue of this research. The following will introduce how such games are designed from the perspective of personalization and adaptation.

### Personalized Design in Games

Personalized game design refers to games tailored for gamers based on their personality characteristics, game abilities, game styles, preferences, etc. (Sedleniece and Cakula, 2012; Aljabali and Ahmad, 2018). The personalized design of the game is mainly divided into four aspects.

1) Personalized virtual characters. Many researchers use different methods to personalize the design of virtual characters in the game, which can significantly increase the immersion and real experience of game players, and stimulate their own cognitive abilities (Reategui et al., 2006; Hollingdale and Greitemeyer, 2013; Zibrek et al., 2018; Peng et al., 2019).
2) Personalized game mode. The first personalization mode is to divide players into different types, and different types of players play different games (Nagle et al., 2016; Orji et al., 2017). The second type of personalization involves recommending different games according to the game players' gaming preferences without having to divide them into different types of players (González et al., 2019).
3) Personalized scene effects and sound effects. We can use visual effects and sound effects to design the personalize features of game. Separate design from the sequence of game scenes and the background music of the game to make the game more attractive and retention rate (Lin et al., 2013; Li et al., 2016).
4) Personalized game content. Personalized game content means that the game content is tailor-made for gamers, is “personalized” and adjusted according to the needs and preferences of specific gamers. Kucirkova et al. personalize the game content in different ways, so that gamers have an excellent gaming experience (Kucirkova and Flewitt, 2020).

### Adaptive Design in Games

Most of the research on adaptive design is mainly reflected in the difficulty adaptation of the game (Mishra et al., 2016). The adaptively designed game will provide game players with continuous and appropriate level challenges, thereby enhancing the player's participation experience in the game (Orvis et al., 2008; Belanich et al., 2013; Csikszentmihalyi, 2014). The adaptive design of the game is mainly divided into three aspects.

1) Change the parameters in the game in real time. Real-time change the number of static objects in the scene, the speed of the character, the attack value of the character, and so on. Studies have shown that the difficulty of the game is balanced with the player's ability, which can stimulate the enthusiasm of the player, effectively master various skills in the game, and enable the player to successfully complete the challenge (Denisova and Cairns, 2019; Peng et al., 2019; Plass et al., 2019).
2) Automatically adjust the game difficulty level for game players. Before the game starts, each game has a corresponding difficulty level. In the game, according to the data generated by the player during the game, the player is automatically matched with a game of suitable difficulty.
3) Dynamically generate new game content. The new content of the game is not generated manually, but automatically generated by the computer through the monitoring of the game data during the game. The game contains help, hints, storyline, etc. Different from personalized game content, adaptively and dynamically generated game content does not completely depend on the player's needs and interests, but is adjusted according to the player's real-time performance in the game.

### Suggestions in Games Design

Combining the above-mentioned personalization and adaptive design of current electronic science games, we put forward the following suggestions for integrating personalization and adaptive design into electronic science games.

1) Early stage of game design. According to personalized design methods, many different types of games can be designed. The game can provide a variety of virtual character models, or design different content for the game (such as game tasks, help content, etc.). According to the adaptive design method, scenes with multiple difficulty levels can be designed for the same game.
2) Data collection and analysis in the game. Collect behavioral data of game players. When necessary, collect physiological signals such as EEG and ECG of game players, and perform real-time pre-processing, feature extraction and classification operations after acquiring the data. The game state is dynamically adjusted according to the data information of the game player.
3) Real-time difficulty adjustment design in the game. According to the previously suggested analysis results, determine whether the current game difficulty matches the player's ability, and then combine the adaptive design method to change the game difficulty so that the game player can get the best gaming experience.

**Figure 1:**
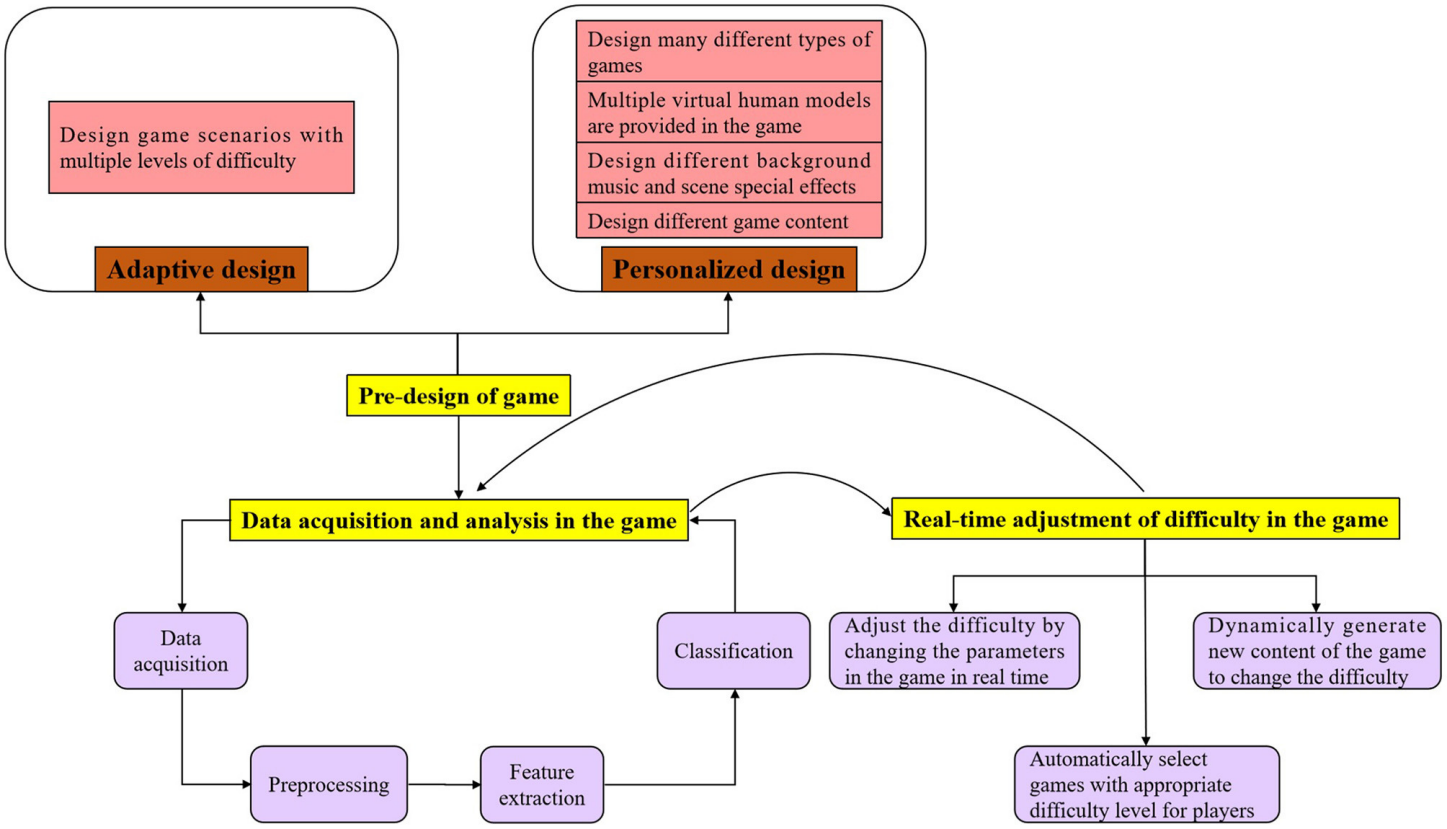
Flow chart of adaptive and personalized game design. “Pre-game design” includes personalized design and adaptive design of the game. After the completion of the pre-game design, we can start “data collection and analysis in the game,” and then adjust the difficulty of the game in real time according to the analysis results. Among them, “data acquisition and analysis in the game” and “real-time adjustment of difficulty in the game” feedback each other.

After completing the pre-design of the game, we can play the game, collect and analyze the physiological signals of the subjects in real time, and then adjust the difficulty of the game in real time according to the analysis results. Among them, the data collection and analysis in the game and the real-time adjustment of the difficulty in the game feedback each other. The specific design process is shown in Figure 1.

## Discussion

In short, electronic science games that integrate personalization and adaptive design to improve cognitive ability can not only stimulate the interest of gamers, but also give gamers a stronger sense of substitution, and can also cultivate their concentration and execution. In terms of cognition, it has higher practicality (Guangxin and Fei, 2006).

However, there are still some problems to be solved in the personalized and adaptive design of electronic science games at this stage.

1) The ability of personalized recommendation needs to be improved. According to existing research, the personalized design concept of games is reflected in the fact that gamers can choose their favorite virtual avatars, game modes, scene effects, and game content, but most of the time gamers cannot distinguish which is their favorite, the result of the choice will cause the training results may not be the most effective.
2) The performance of data analysis algorithms needs to be improved. In the game, when the difficulty of the game is dynamically adjusted according to the player's state, it is necessary to collect player behavior data, and dynamically change the difficulty of the game according to the results of data analysis. In order to obtain the best adaptation effect, better data analysis algorithms are needed.
3) The player's immersive experience needs to be improved. Although the personalized and adaptive design of the game has been achieved at this stage, some players still have problems such as inattention, so next you can consider integrating the game into virtual reality to enhance their immersive experience.

Therefore, with the continuous development of electronic science games, we need to overcome the above-mentioned challenges and contribute to their further application in cognitive enhancement. In addition, considering that Alzheimer's Disease patients have obvious cognitive decline, it is a good choice to improve their cognitive ability to use the electronic science games that embody personalization and adaptability in the future.

## Author Contributions

YZhou, DW, and WY contributed to conception and design of the study. WY, YZhon, and HC searched the database. DW, WY, YZhon, and HC performed the analysis of literatures. WY, JX, and DW wrote the first draft of the manuscript. SW, YZhon, and HC wrote sections of the manuscript. XD and MS revised this paper and analyzed the literatures. All authors contributed to manuscript revision, read, and approved the submitted version.

## Funding

This work was supported in part by National Natural Science Foundation of China (61876165 and 61503326), Natural Science Foundation of Hebei Province in China (F2016203343).

## Conflict of Interest

The authors declare that the research was conducted in the absence of any commercial or financial relationships that could be construed as a potential conflict of interest.

## Publisher's Note

All claims expressed in this article are solely those of the authors and do not necessarily represent those of their affiliated organizations, or those of the publisher, the editors and the reviewers. Any product that may be evaluated in this article, or claim that may be made by its manufacturer, is not guaranteed or endorsed by the publisher.
